# Age of the Domestic Animals

**Published:** 1892-02

**Authors:** 


### BOOK NOTICES.

Age of the Domestic Animals. Being a complete treatise on the dentition of the horse, ox, sheep, hog, and dog, and of the various other means of determining the age of these animals. By Rush Shippen Huidekoper, M. D., late Dean of the Veterinary Department, University of Pennsylvania. Philadelphia (1231 Filbert St.) and London, the F. A. Davis Co., 1891. Complete in one handsome royal octavo volume of 225 pages, bound in extra cloth. Price, in United States and Canada, post-paid, $1.75 net; Great Britain, 10s.; France, 12 fr. 20.

This work, fully illustrated, will fill the long-felt need of a practical text-book of the ordinary subjects pertaining to domestic animals. The author has drawn freely in quotations from French, German, and Italian works. The age of the domesticated animal is a matter of great importance in agricultural commerce, as in the limited period during which each of them is individually useful, a comparatively short line diminishes greatly the extent of usefulness to which each can be put, and consequently lowers its value as an investment. This book is invaluable to owners of animals and especially to physicians as an aid in purchasing horses. All should have one.

